# Chimeric Antigen Receptor-T-Cell Therapy for B-Cell Hematological Malignancies: An Update of the Pivotal Clinical Trial Data

**DOI:** 10.3390/pharmaceutics12020194

**Published:** 2020-02-24

**Authors:** Gils Roex, Tom Feys, Yves Beguin, Tessa Kerre, Xavier Poiré, Philippe Lewalle, Peter Vandenberghe, Dominique Bron, Sébastien Anguille

**Affiliations:** 1 Tumor Immunology Group, Laboratory of Experimental Hematology, Vaccine & Infectious Disease Institute (VAXINFECTIO), University of Antwerp, 2650 Edegem, Belgium; gils.roex@uantwerpen.be; 2 Ariez International BV, 9000 Ghent, Belgium; t.feys@ariez.com; 3 Department of Hematology, University of Liège, 4000 Liège, Belgium; yves.beguin@chu.ulg.ac.be; 4 Department of Hematology, University Hospital Ghent, 9000 Ghent, Belgium; tessa.kerre@ugent.be; 5 Faculty of Medicine and Dentistry, Université Catholique de Louvain, 1200 Woluwe-Saint-Lambert, Belgium; xavier.poire@uclouvain.be; 6 Department of Hematology, Institut Jules Bordet, 1000 Brussels, Belgium; philippe.lewalle@bordet.be (P.L.); dominique.bron@bordet.be (D.B.); 7 Department of Hematology, University Hospitals Leuven, 3000 Leuven, Belgium; peter.vandenberghe@uzleuven.be; 8 Center for Cell Therapy & Regenerative Medicine (CCRG) and Division of Hematology, Antwerp University Hospital, 2650 Edegem, Belgium

**Keywords:** CAR-T cells, immunotherapy, B-cell malignancies, CD19, BCMA

## Abstract

Chimeric antigen receptor (CAR)-T-cell therapy is an innovative form of adoptive cell therapy that has revolutionized the treatment of certain hematological malignancies, including B-cell non-Hodgkin lymphoma (NHL) and B-cell acute lymphoblastic leukemia (ALL). The treatment is currently also being studied in other B-cell neoplasms, including multiple myeloma (MM) and chronic lymphocytic leukemia (CLL). CD19 and B-cell maturation antigen (BCMA) have been the most popular target antigens for CAR-T-cell immunotherapy of these malignancies. This review will discuss the efficacy and toxicity data from the pivotal clinical studies of CD19- and BCMA-targeted CAR-T-cell therapies in relapsed/refractory B-cell malignancies (NHL, ALL, CLL) and MM, respectively.

## 1. Introduction

For decades, the treatment of hematological malignancies was dominated by systemic chemotherapy, radiation therapy, and stem cell transplantation. More recently, new insights in the genetic and molecular basis of these malignancies paved the way for the development of targeted therapies, while the increased understanding of the interplay between the patient’s immune system and cancer cells led to the development of several innovative immunotherapies. One of these immunology-based treatment strategies that recently generated much excitement is chimeric antigen receptor (CAR)-T-cell therapy [1]. This type of adoptive cell therapy (ACT) already proved to be a real breakthrough in the treatment of certain non-Hodgkin lymphoma (NHL) types and B-cell acute lymphoblastic leukemia (ALL), and is currently also being evaluated in other hematological malignancies, including multiple myeloma (MM) and chronic lymphocytic leukemia (CLL) [1].

Exploiting the immune system to attack cancer cells is not a new concept. In fact, the development of allogeneic stem cell transplantation (alloSCT) has first highlighted the potential of T cells to eliminate cancer cells. In this respect, Kolb et al. showed that donor lymphocyte infusions can induce long-lasting remissions in patients with relapsed chronic myeloid leukemia (CML) [2]. With ACT, immune cells are collected from a patient or a donor after which they are manipulated and/or expanded ex vivo and reinfused to the patient [1]. The success of ACT mainly depends on the presence of an adequate amount of effector cells in the patient, which in turn requires precursors with either natural anti-tumor recognition, or engineering of T cells to provide this recognition [1]. Therefore, researchers have developed several strategies to improve the tumor recognition of adoptively stimulated cells. Genetic engineering of novel receptors (i.e., CARs) led to the development of molecules that can both recognize proteins present on the surface of tumor cells and provide T-cell activation, proliferation, and memory [3]. CAR constructs are hybrid molecules; the extracellular part is based on the structure of a monoclonal antibody and responsible for surface antigen recognition. This recognition occurs in a major histocompatibility complex (MHC)-independent manner. The intracellular part is based on the structure of the T-cell receptor (TCR) coupled with one or more co-stimulatory domains, allowing to transduce the antigen recognition into T-cell activation [3].

## 2. CAR-T-Cell Design

In general, CARs are composed of three major domains: an ectodomain, a transmembrane domain, and an endodomain. The ectodomain or extracellular portion of the CAR typically consists of heavy and light chains derived from an antibody in single-chain variable fragment format, and a hinge region. It redirects the specificity of the receptor to recognize antigens on the cell surface independently of MHC molecules. CD19 has been most frequently chosen as target antigen in B-NHL, B-ALL, and B-CLL for several reasons: its frequent and high-level expression in these malignancies, with a broader and higher expression relative to other potential targets like CD20 or CD22, and its restriction to the B-cell lineage in healthy tissue. The transmembrane domain of the CAR construct primarily plays a role in stabilizing the CAR, while the intracellular endodomain provides the necessary signals to activate the T cells after antigen recognition [3].

The design of CARs considerably evolved over the years. First-generation CARs were designed similarly to the endogenous TCR complex. In these initial constructs, the intracellular component usually consisted of CD3ζ, which was linked to an extracellular antigen-recognition domain that allowed for direct, MHC-independent recognition of antigens on the tumor cell surface [4]. Importantly, these first-generation designs did not include co-stimulatory domains and, as such, did not provide a second signal for full T-cell activation. As a result, these first-generation CAR-T cells were more prone to apoptosis and had limited in vivo expansion potential, resulting in poor cytotoxicity [4]. The addition of co-stimulatory signaling domains (e.g., CD28, 4-1BB) in second-generation CARs resulted in improved T-cell activation, enhanced survival capabilities, and a more effective expansion of the modified T cells in vivo [4,5]. These second-generation receptors form the basis of the currently approved CAR-T-cell therapies. It is now becoming increasingly clear that each type of co-stimulatory domain has specific roles in CAR signaling; for example, CD28-based CAR-T cells exhibit more potent effector cell functions but limited persistence, whereas 4-1BB tends to drive the CAR-T cells towards a central memory phenotype resulting in improved persistence [6,7]. Third-generation CAR-T cells combine the signaling potential of two co-stimulatory domains (e.g., both CD28 and 4-1BB). The anti-tumor activity of fourth-generation CARs, including T cells redirected for universal cytokine-mediated killing (TRUCKs), is even further enhanced by additional genetic modifications, for example by the addition of transgenes for cytokine secretion (e.g., IL-12) [8,9].

## 3. CAR-T-Cell Manufacturing and Administration

**Figure 1:**
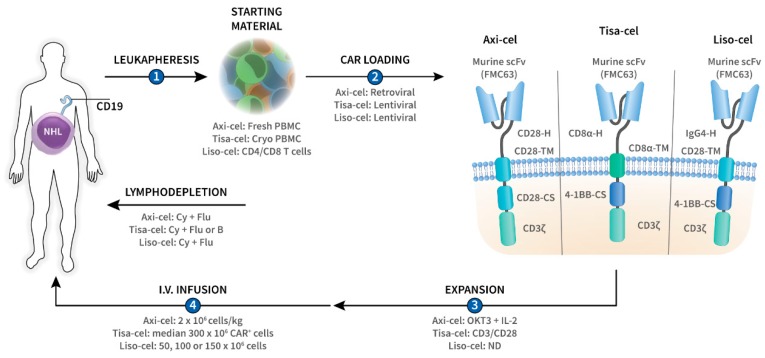
Overview of CD19-targeted chimeric antigen receptor (CAR)-T-cell therapies axicabtagene ciloleucel (axi-cel), tisagenlecleucel (tisa-cel), and lisocabtagene maraleucel (liso-cel) in CD19^+^ non-Hodgkin lymphoma (NHL). T cells are collected from the patient by leukapheresis (**1**) after which they are loaded with the *CD19 CAR* gene by means of lentiviral or retroviral transduction (**2**), and ex vivo expanded (**3**). The resultant CAR-T cells are then administered back to the patient by intravenous (i.v.) infusion (**4**). Lymphodepleting chemotherapy is usually administered prior to CAR-T-cell infusion in order to promote in vivo CAR-T-cell expansion and persistence. Axi-cel, tisa-cel, and liso-cel are second-generation CARs, of which the intracellular part contains the T-cell receptor ζ chain (CD3ζ) and a co-stimulatory (-CS) domain (CD28 or 4-1BB). The intracellular part is linked by the transmembrane domain (-TM) with the extracellular part of the CAR which is composed of the hinge and the antigen-recognition domain. The three constructs bear a different hinge (-H) but share the same murine FMC63-derived single chain variable fragment (scFv) as antigen-binding domain. B, bendamustine; CD3/CD28, anti-CD3/CD28 microbeads; Cy, cyclophosphamide; Flu, fludarabine; IL-2, interleukin-2; ND, no data; OKT3, anti-CD3 monoclonal antibody; PBMC, peripheral blood mononuclear cells.

Although allogeneic CAR-T cells have been used, the production of CAR-T cells typically starts with the collection of peripheral blood mononuclear cells (PBMCs) from the patient (autologous) using a large volume leukapheresis procedure (Figure 1). The cells are then transferred to a cell-processing facility where they are loaded with the CAR, usually by incubating them with CAR-encoding viral vectors, which enter the T cells and introduce the *CAR* RNA (Figure 1). This *CAR* RNA is then reverse transcribed into DNA, which recombines into the T-cell genome, resulting in permanent *CAR* gene incorporation. Both lentiviral and, to a lesser extent, gamma-retroviral vectors have been used for *CAR* gene transduction of primary T cells (Figure 1) [10].

The *CAR* gene-modified T cells are then expanded ex vivo and prepared as a pharmaceutical intravenous infusion product. The cells are usually administered as single infusion. The median time from leukapheresis to CAR-T-cell administration is 4–5 weeks and the entire process from referral to infusion can take up to 2 months [11]. Therefore, physicians often perform bridging chemotherapy to avoid rapid disease progression and to maintain the patient’s general condition during the CAR-T-cell production period. Lymphodepleting (LD) chemotherapy, such as fludarabine and cyclophosphamide, is often administered prior to the infusion of the CAR-T cells (Figure 1) [12]. LD chemotherapy decreases the number of T cells in vivo, including regulatory T cells, and consequently upregulates cytokines such as IL-7 and IL-15 [12]. These cytokines promote T-cell expansion and augment the anti-tumor activity of the CAR-T cells.

## 4. Efficacy and Toxicity of CAR-T-Cell Therapy in B-Cell Malignancies

CAR-T-cell therapy has emerged rapidly over the last few years, ultimately leading to the approval of the first two CAR-T-cell medicines, tisagenlecleucel (tisa-cel) and axicabtagene ciloleucel (axi-cel) both by the US Food and Drug Administration (FDA) and later by the European Medicines Agency (EMA) for the treatment of certain B-cell NHL types in adults, as well as relapsed/refractory (r/r) B-ALL in children and young adults. In addition to this, the potential of CAR-T-cell therapy is also being explored in other B-cell neoplasms, such as MM and B-CLL [1,8].

### 4.1. Non-Hodgkin Lymphoma

B-cell NHL is the most frequent hematological malignancy, with diffuse large B-cell lymphoma (DLBCL) being the most common subtype. Despite therapeutic improvements, a substantial proportion of DLBCL patients develop chemorefractory disease. Currently, approximately two-thirds of patients with newly diagnosed DLBCL are cured with first-line cyclophosphamide, doxorubicin, vincristine, and prednisolone (CHOP) therapy in combination with rituximab [13]. The standard of care second-line treatment for fit patients with r/r DLBCL is salvage chemotherapy followed by autologous SCT (ASCT). Unfortunately, approximately half of the patients will remain refractory or experience a relapse after second-line treatment [13]. Relapsed/refractory DLBCL faces a grim prognosis; based on data from the SCHOLAR-1 study, a multicohort, retrospective study involving 636 patients with pooled data from two phase III studies (CORAL and LY.12) and two observational cohorts, the median overall survival (OS) for patients with r/r DLBCL is only 6.3 months (95% CI: 5.9–7.0 months) [14]. To overcome this chemorefractoriness in DLBCL, several novel therapeutic strategies have been explored, including CAR-T-cell therapy. Several early, single-center studies demonstrated significant anti-lymphoma activity of CD19-directed CAR-T-cell therapy in NHL patients and formed the basis for the design of three larger multicenter clinical trials [15,16].

The phase II portion of the ZUMA-1 trial evaluated axi-cel in patients with refractory, high-grade B-cell lymphoma. In this study, no bridging therapy was allowed, and the LD regimen consisted of cyclophosphamide and fludarabine. Patients in the trial were divided in two cohorts: cohort 1—the largest cohort—included DLBCL patients, while cohort 2 consisted of patients with transformed follicular lymphoma (TFL) and primary mediastinal B-cell lymphoma (PMBCL) [17,18]. The primary endpoint in ZUMA-1 was overall response rate (ORR) in patients with more than 6 months follow-up after axi-cel infusion, as compared with historical control (SCHOLAR-1 [14]). In total, 111 patients were enrolled of whom 101 received axi-cel. More than two-thirds of the patients were refractory to at least three lines of therapy and 21% relapsed within 12 months after an ASCT. In the most recent report of this trial, with a median follow-up of 27.1 months, an ORR of 83% was demonstrated with a complete remission (CR) rate of 58% [17]. This represents an eightfold higher CR rate in comparison with SCHOLAR-1 [14]. The median duration of response is still not reached for patients with a CR (95% CI: 12.9 months–not estimable), underscoring the durability of the responses to axi-cel [17]. A more detailed overview of the efficacy data in ZUMA-1 can be found in Table 1 [17,18].

The JULIET trial was a phase II multicenter global study in patients with r/r B-cell NHL using the anti-CD19 CAR-T-cell product tisa-cel [19,20]. Key eligibility criteria in JULIET included aggressive B-cell lymphoma (DLBCL, representing 80% of the treated patients, or TFL); about half of the patients had refractory disease with at least three prior lines of therapy (including ASCT in 49% of the patients). In contrast to ZUMA-1, cryopreserved apheresis products were utilized, and bridging chemotherapy was allowed for patients with rapidly progressive disease [20]. Overall, 92% of the patients received bridging chemotherapy. LD chemotherapy consisted of cyclophosphamide and fludarabine, or bendamustine. Similar to the ZUMA-1 trial, the primary endpoints of the trial were ORR and the rate of CR. A total of 165 patients were enrolled and 111 patients were infused with tisa-cel. In the 93 response-evaluable patients (at least 3 months of follow-up), the reported ORR and CR rates were 52% and 40%, respectively. More efficacy details are shown in Table 1 [19].

Based on the promising results of ZUMA-1 and JULIET, the US FDA approved axi-cel and tisa-cel for certain r/r B-cell NHL subtypes in October 2017 and May 2018, respectively. A couple of months later, both agents were also approved by the EMA. With the approval of axi-cel and tisa-cel, interest in reporting the efficacy of this therapy in real clinical practice grew. “Real-world” data on the use of axi-cel were reported by Nastoupil et al. [24], Jacobson et al. [25], and others [26]. Overall, 43% of the patients in the study by Nastoupil et al. did not meet the inclusion criteria of ZUMA-1. Moreover, 55% received bridging therapy whereas this was not allowed in ZUMA-1. Of the 294 leukapheresed patients, 274 were actually infused. Best ORR (81%) and CR (57%) rates were similar to those reported in ZUMA-1 (83% and 58%, respectively). This essentially confirms that the efficacy of axi-cel in r/r B-cell NHL (including DLBCL, TFL, and PMBCL) could be replicated outside the strict eligibility criteria of clinical trials [24,25,26].

The multicenter TRANSCEND NHL 001 study of lisocabtagene maraleucel (liso-cel) is the largest CD19 CAR-T cell study performed so far; 344 patients with a variety of r/r B-cell NHL types, including DLBCL, TFL, PMBCL, FL grade 3b, and other high-grade B-cell lymphomas, were leukapheresed [21,22,23]. Like in ZUMA-1 and JULIET, DLBCL was the most common histological subtype. Bridging therapy was allowed and required in approximately two-thirds of the patients. A cyclophosphamide and fludarabine combination was used for lymphodepletion. In total, 294 patients were infused in this trial, but 25 patients received a nonconforming product. The best ORR and CR rates among the 256 response-evaluable patients were 73% and 53%, respectively [21]. The PFS and OS data are presented in Table 1 [21].

The most common acute toxicities observed after CAR-T-cell therapy are CRS and immune effector cell-associated neurotoxicity syndrome (ICANS, previously termed CAR-T-cell-related encephalopathy syndrome (CRES)), either of which can be lethal [27]. CRS is caused by cytokine elevations as a result of immune activation of large numbers of lymphocytes. The cardinal symptoms include fever, hypotension, and hypoxemia [27]. The median time to onset of CRS was 2–3 days with axi-cel in ZUMA-1 [17,18] and tisa-cel in JULIET [19], and 5 days with liso-cel in TRANSCEND [21]. In recent years, guidelines for the uniform grading of CRS have been published, of which the guidelines by the American Society for Transplantation and Cellular Therapy (ASTCT) have become the most widely adopted [28]. CRS is graded with a score of 1 (mild) to 4 (life-threatening) [28]. In ZUMA-1 (axi-cel) [17,18], JULIET (tisa-cel) [19], and TRANSCEND (liso-cel) [21], the incidence of any grade CRS was 92%, 58%, and 42%, respectively (Table 2). Grade ≥3 CRS occurred in 11%, 22%, and 2%, respectively (Table 2). In the real-world study by Nastoupil et al., 7% of the patients developed severe CRS [24,26].

Interleukin-6 (IL-6) has been implicated as a central mediator of CRS [27]. This explains why tocilizumab, a therapeutic antibody blocking IL-6 receptors, has become the drug of choice for the management of moderate to severe CRS [28,29]. It induces near-immediate reversal of CRS symptoms in most patients. Importantly, tocilizumab does not seem to affect the efficacy of CAR-T-cell therapy in terms of ORR, CR rate, or the durability of responses [29]. In ZUMA-1 (axi-cel) [17,18], JULIET (tisa-cel) [19] and TRANSCEND (liso-cel) [21], tocilizumab was used in 43%, 14%, and 19% of the patients, respectively (Table 2). In the real world, tocilizumab is far more frequently used (in 63% of the cases in the study with axi-cel by Nastoupil et al.) [24,25,26]. Until recently, corticosteroids were used only in severe CRS cases due to concerns regarding their suppressive action on T-cell function [29]. However, it is becoming increasingly clear that corticosteroids can be used safely to treat CAR-T-cell-related toxicities without limiting efficacy. This statement is further strengthened by the real-world data on the use of axi-cel in r/r B-cell NHL (i.e., similar efficacy in ZUMA-1 and real-world study by Nastoupil et al., despite the proportionally higher use of corticosteroids to treat CRS (55% vs. 27% in ZUMA-1)) [24,26].

Neurotoxicity, termed ICANS or CRES, is the second most common serious adverse reaction after administration of CAR-T-cell therapy [28]. Affected patients develop toxic encephalopathy with confusion, aphasia, ataxia, delirium, seizures, and cerebral edema [28]. The causative pathophysiology of these neurological side effects is still not fully understood. IL-6 does not seem to play an important role in ICANS/CRES; in mouse models, it was elegantly shown that anti-IL-6 therapy with tocilizumab did not have a major impact on the development and evolution of ICANS/CRES [30]. Nevertheless, tocilizumab will often be used, especially if the neurotoxicity co-occurs with CRS. Otherwise, corticosteroids are the preferred treatment or, if available, the IL-1 blocker anakinra. The severity of ICANS can fluctuate rapidly, necessitating close patient monitoring. This is especially important for the very rare, but life-threatening cerebral edema, for which anti-IL-6 therapy is not effective [29]. Similar to CRS, management of ICANS is based on the severity of the neurological symptoms. The 10-point “Immune Effector Cell-Associated Encephalopathy (ICE)” scoring tool is now the gold standard for screening and grading of ICANS [28]. Neurotoxicity appears to be more common with axi-cel (67% with 32% grade ≥3 in ZUMA-1 [17,18]), as compared to tisa-cel (21% with 12% grade ≥3 in JULIET [19]) and liso-cel (30% with 10% grade ≥3 in TRANSCEND [21]) (Table 2).

### 4.2. B-Cell Acute Lymphoblastic Leukemia

The phase II ELIANA trial investigated the CD19-directed genetically modified autologous T-cell product tisa-cel as a single infusion for r/r pediatric and young adult B-cell ALL [31]. From the 107 patients who were screened, 92 were enrolled; 17 patients could not be infused for a variety of reasons: death (N = 7), serious adverse events (N = 3) or CAR-T-cell production failure (N = 7). Of the 75 tisa-cel-treated patients, 65 (87%) required bridging chemotherapy between enrolment and infusion, and 72 (96%) received LD chemotherapy (mostly fludarabine plus cyclophosphamide). Patients in the study received a median of three prior therapies, and 61% of patients previously underwent an alloSCT. The CR rate at 3 months was 81% and the median duration of the remission was not reached with a median follow-up of 1 year. All patients with a treatment response were negative for minimal residual disease (MRD). The event-free survival (EFS) and OS rates at 6 months were 73% and 90%, respectively, dropping to 50% and 76% at the 1-year landmark [31]. Long-term in vivo persistence was demonstrated. All patients with a response to treatment had B-cell aplasia, and most patients in the study received immunoglobulin replacement in accordance with local practice. Grade 3/4 adverse events (AEs) with a suspected relation to tisa-cel occurred in 73% of patients. CRS occurred in 77% of patients, of whom 48% received tocilizumab. Neurotoxicity was observed in 40% of patients; all these events took place within the first 2 months [31]. Tisa-cel has received regulatory approval for the treatment of pediatric and young adult patients up to 25 years of age with B-ALL that is refractory, in relapse after alloSCT or in second or later relapse.

### 4.3. Multiple Myeloma

Multiple myeloma is a B-cell neoplasm characterized by a malignant proliferation of plasma cells in the bone marrow. Over the last decade, we have witnessed enormous progress in the treatment of MM, but despite these advances, the disease remains incurable. Therefore, the development of new therapeutic drugs is needed, and CAR-T-cell therapy is considered promising. B-cell maturation antigen (BCMA) is the most widely used target antigen in CAR-T-cell studies for MM [32,33,34]. BCMA expression is largely restricted to (malignant) plasma cells and some mature B cells [35,36]. BCMA appears to play an important role in the promotion of MM cell survival, proliferation, and was also found to be involved in the development of drug resistance [37]. Table 3 provides an overview of all BCMA CAR-T-cell clinical trials in MM that were published as full article on Web of Science/Pubmed (date of last search: 01 Jan 2020) [38,39,40,41,42,43,44]. Due to the early phase character of most trials, the number of infused patients was rather low. The ORR was in the range of 85–95% in most studies; only two studies, NCT02546167 [38] and NCT02215967 [39,40], reported lower ORR and CR rates. Possible explanations are the suboptimal BCMA CAR-T cell doses that were used in these trials and the fact that most patients were heavily pretreated. The median PFS observed with BCMA CAR-T-cell therapy was in the range of 1 year [41,42,43,44]. As shown in Table 3, most patients developed CRS; grade 3 or higher CRS was observed in 5–41% of the patients. Neurotoxicity was an uncommon event, usually occurring in less than 10% of the patients. Only two studies reported neurotoxicity rates of 32% [38%] and 42% [41].

Despite the relatively high ORR obtained with BCMA CAR-T-cell therapy, the observed therapeutic effect was often transient and relapses were frequently observed. Downregulation or loss of BCMA expression is likely an important mechanism underlying these relapses [45]. Therefore, targets other than BCMA, such as CD19 or CD138, have been investigated in CAR-T-cell studies, but yielded varying results [46,47]. Dual antigen targeting, for example by combining BCMA and CD19 CAR-T cells, is also being pursued in an attempt to improve response durability [44]. CD19 is a rather unconventional target antigen in MM, because myeloma cells are mostly CD19-negative by flow cytometry. Nevertheless, more sensitive techniques have recently revealed that CD19 is expressed at ultra-low levels on MM cells, and that these levels are sufficient for recognition of MM cells by CD19 CAR T-cells [48]. Moreover, it appears that CD19^+^ MM cells bear features of a cancer stem cell (i.e., self-renewal and drug resistance), making it an attractive target for immunotherapy [49]. Another strategy to avoid BCMA-negative relapses involves the combination of BCMA CAR-T cells with gamma-secretase inhibitors which prevent cleavage of BCMA from the MM cell surface [50]. In addition to this, other studies are looking into the potential of CAR T-cell therapies targeting other antigens, including CD38, SLAMF7, CD44v6, CD56, GPRC5D, amongst others [51]. There are currently no CAR-T-cell therapies for MM that have received regulatory approval yet, but the first approvals are expected later this year or in 2021.

### 4.4. Chronic Lymphocytic Leukemia

B-CLL was one of the first diseases in which CD19 CAR-T cells were tested. Since the first report of the efficacy of second-generation CAR-T cells against CLL in 2011 [52], results have been reported of CD19-targeted CAR-T-cell therapy in a total of 134 CLL patients [53]. Overall, the CLL patients who were treated with CAR-T-cell therapy had a particularly poor prognosis, with most of them being in relapse after a large number of treatment lines. In total, 74 of the 108 (68.5%) evaluated patients in these studies had p53 alterations, and 41 out of 70 (58.6%) had a complex karyotype [53]. A second observation from the different CAR-T-cell reports in CLL is that the efficacy is lower for CLL than for DLBCL or B-ALL: CR, according to the IWCLL criteria, was obtained in only a minority (20–30%) of patients with an estimated 18-month PFS of 25% [54,55,56]. Interestingly, responses appeared to be weaker in the lymph nodes than in the bone marrow and blood. In fact, in some series, a substantial proportion of patients treated with CAR-T cells obtained undetectable MRD in the bone marrow [55,57,58]. For example, in a study by Turtle et al. including 24 r/r CLL patients who previously received ibrutinib, an ORR of 71% (21% CR) was reported four weeks after the CAR-T-cell infusion, with bone marrow negativity in 58%. Among these MRD negative patients, the PFS and OS rate was almost 100% at a median follow-up of 6.6 months [55].

The lower efficacy of CAR-T cells in CLL may be partly due T-cell exhaustion in CLL patients resulting in decreased CAR-T-cell functionality [59]. To overcome this, several research groups are looking into ways to optimize the CAR constructs in CLL. In addition to this, studies are underway looking into the potential of combining CAR-T-cell therapy with other anti-CLL therapies. In this respect, data suggest that ibrutinib may improve the outcome in CLL patients receiving CAR-T cells [57,58]. Based on these observations, a prospective study will further evaluate the efficacy of ibrutinib maintenance at the time of injection of the CAR-T cells (NCT03331198).

## 5. Conclusions and Future Perspectives

CAR-T-cell therapy is becoming an important addition to the treatment of r/r B-cell malignancies [60]. CD19-targeted CAR-T-cell therapies have shown unprecedented clinical activity in certain aggressive B-cell NHL subtypes, including DLBCL. The three most advanced CD19 CAR-T-cell products for use in NHL are axi-cel, tisa-cel, and liso-cel; the first two have already received FDA and EMA approval and are now reimbursed in several countries [60]. In the absence of head-to-head clinical trial data, it is not possible to directly compare the effectiveness of these three agents. Nevertheless, the overall CR rate lies in the range of 50%, which is indeed exceptionally high in patients with chemorefractory DLBCL who have failed several prior lines of therapy [14]. Moreover, PFS curves for these three agents show a plateau at their tail, indicating that durable responses can be observed in approximately 1/3 NHL patients [17,19]. This high efficacy, however, comes at a cost of substantial toxicity. Based on the toxicity data presented in this review (Table 2), it can be concluded that liso-cel has a favorable safety profile in terms of severe CRS and neurotoxicity [21] but whether this is product-dependent remains to be determined [61]. In B-ALL, tisa-cel is the only CD19 CAR-T-cell product that has received regulatory approval so far. It is marketed for the treatment of pediatric and young adult patients up to 25 years of age with r/r B-ALL. Toxicity is considerable, but generally accepted given the very few effective salvage treatment options available for these patients [31]. Patients with r/r MM can benefit from BCMA-targeted CAR-T-cell therapy. BCMA CAR-T cells are highly active in r/r MM, with ORRs of 85–95% (Table 3) and CR rates of up to 80% in selected studies [42]. The median PFS is around 12 months, which is also unprecedently high in heavily pretreated MM patients. Toxicity is common, with CRS reported in >75% of the patients. The occurrence of neurotoxicity appears to be product-specific (Table 3). Finally, in r/r B-CLL, CD19 CAR-T cells have been tested but response rates were rather disappointing [54,55,56]. In these patients, combination strategies with, for example, ibrutinib, may be required to unlock the full therapeutic potential of CD19 CAR-T-cell therapies [62].

Concerning efficacy, the focus must now be placed on improving response durability and thus also on developing strategies to tackle relapse. Two main mechanisms of relapse following CAR-T-cell therapy have been identified, including relapses due to loss or downregulation of the target antigen (antigen-negative relapses) and relapses due to poor persistence and exhaustion of the CAR-T cells (so-called antigen-positive relapses because the target antigen is still retained on the tumor cell surface) [63]. Antigen-negative relapses can be caused by selective pressure of the CAR-T cells on the tumor cells, resulting in outgrowth of antigen-negative clones or clones with reduced antigen expression [64]. Fry et al. were the first to establish that r/r B-ALL patients experiencing an antigen-negative relapse after CD19-targeted CAR-T-cell therapy can be rescued by using CAR-T cells targeting an alternative antigen: CD22 [65]. This has fueled the development of dual antigen-targeted approaches to overcome antigen escape [66]. Several early-phase CAR-T-cell clinical trials investigating the combined targeting of CD19 and another antigen, such as CD22 and CD20, have now been initiated in patients with CD19^+^ B-cell malignancies [67,68]. Similarly, in MM, a dual antigen approach with BCMA and CD19 CAR-T cells has already been published [44], and novel MM antigens (e.g., GPRC5D) are being identified at rapid pace for rational combined targeting with BCMA [51,69]. In MM, BCMA-negative relapses can also be prevented by the combined use of BCMA CAR-T cells and an inhibitor of γ-secretase, an enzyme responsible for active cleavage of BCMA from the MM cell surface [50].

Antigen-positive relapses can be overcome by improving persistence and by putting anti-exhaustion measures in place. One of the strategies is to use a low-affinity CD19 CAR (CAT) with a faster CD19 interaction time than the scFv from FMC63, which is the anti-CD19 antibody used in axi-cel, tisa-cel, and liso-cel (Figure 1). In a small cohort of pediatric r/r B-ALL patients (n = 14), prolonged CAR-T-cell persistence was observed with the use of this CAT CAR with durable responses [70]. Tonic signaling, i.e., constitutive CAR triggering in the absence of the target antigen, has been recognized as an important mechanism leading to CAR-T-cell exhaustion [9,71]. Long et al. showed that the choice of co-stimulatory domain has an impact on this phenomenon, with CD28 augmenting and 4-1BB mitigating CAR-T-cell exhaustion following tonic CAR signaling [7]. A direct pairwise comparison of CD28 and 4-1BB co-stimulated CD19 CAR-T cells in NHL patients revealed that the 4-1BB variant results in improved persistence, indicating that 4-1BB co-stimulation favors more durable responses [72]. Nevertheless, the CD28 co-stimulatory domain may be required in the setting of low CD19 antigen density because CD28 co-stimulated CAR-T cells are far more efficient at targeting CD19-low tumor cells as compared to their 4-1BB counterparts [73]. A growing body of evidence indicates that CAR-T cells from non-responders or (early) relapsers are more prone to exhaustion and display increased expression of immune checkpoint molecules, such as PD-1 [56,74]. Conceptually, immune checkpoint blockade could help to restore the function of these exhausted CAR-T cells and several studies combining CAR-T cells with checkpoint inhibitors are now underway [75,76,77]. To avoid the toxicities of systemically administered checkpoint inhibitors, CAR-T cells have also been genetically modified to locally release a PD-1 blocking antibody [78]. Alternatively, CAR-T cells can also be “armored” with c-Jun to prevent their exhaustion [71].

Concerning toxicity, several approaches are being explored to improve the overall safety profile of CAR-T-cell therapy. Tocilizumab and corticosteroids are now being used when early signs of CRS appear, leading to a decreased incidence of severe CRS [79]. Other strategies involve the incorporation of suicide genes in the CAR construct that can be activated in the event of uncontrolled toxicity [80]. Likewise, CAR-T cells can also be modified to co-express a truncated (inactive) epidermal growth factor receptor (EGFR); use of the anti-EGFR mAb cetuximab will then allow selective depletion of the CAR-T cells in case of severe toxicity [80]. The drawback of these strategies is that they result in an irreversible elimination of the CAR-T cells. Recently, it was shown that the tyrosine kinase inhibitor dasatinib can be used as a pharmacologic “on/off” switch for CAR-T cells; it allows for an immediate and titratable inhibition of the CAR-T cells with a complete restoration of their function upon withdrawal of the drug [81]. Alternatively, when CAR-T cells are transiently modified using CAR-encoding mRNA, potential toxicities will be self-limiting [82], making this approach especially useful for evaluating safety of novel CAR constructs.

Despite these remaining challenges both on the level of efficacy, in particular with respect to improving the response durability, and on the level of toxicity, it is clear that CAR-T-cell therapy is here to stay as an important therapeutic modality for patients with r/r B-cell malignancies.

## Figures and Tables

**Table 1:** Efficacy data of CD19-targeted CAR-T-cell therapies axi-cel, tisa-cel, and liso-cel in NHL.

Cell Product	axi-cel	tisa-cel	liso-cel
Trial [ref.]	ZUMA-1 [17,18]	JULIET [19,20]	TRANSCEND [21,22,23]
**N enrolled (infused)**	111 (101)	165 (111)	344 (269 + 25 ^#^)
**N response-evaluable**	101	93	256
**Best ORR (CR)**	83% (58%)	52% (40%)	73% (53%)
**Median DoR**	11.1 mo (*4.2 mo-n.e.*)	Not reached (*10.0 mo-n.e.*)	Not reached (*8.6 mo-n.e.*)
**Median PFS**	5.9 mo (*3.3–15.0 mo*)	NR	6.8 mo (*3.3–14.1 mo*)
**PFS rate**	24-mo PFS: 72% (*for pts in CR at 3 mo*)	12-mo PFS: 83% (*for pts in CR/PR at 3 mo*)	12-mo PFS: 65% (*for pts in CR*)
**Median OS**	Not reached (*12.8 mo-n.e.*)	12.0 mo ^§^ (*7.0 mo-n.e.*)	21.1 mo (*13.3 mo-n.e.*)
**OS rate**	Est. 24-mo OS: 50.5%	Est. 12-mo OS: 49% (*90% for pts in CR*)	Est. 12-mo OS: 58% (*86% for pts in CR*)

Axi-cel, axicabtagene ciloleucel; CR, complete response; DoR, duration of response; Est., estimated; liso-cel, lisocabtagene maraleucel; mo, months; N, number; n.e., not estimable; NHL, non-Hodgkin lymphoma; NR, not reported; ORR, objective response rate; OS, overall survival; PFS, progression-free survival; PR, partial response; ref., reference; ^#^, number of patients that received a nonconforming product (N = 25); ^§^, median OS reported for the infused population (N = 111).

**Table 2:** Toxicity data of CD19-targeted CAR-T-cell therapies axi-cel, tisa-cel, and liso-cel in NHL.

Cell Product	axi-cel	tisa-cel	liso-cel
Trial [ref.]	ZUMA-1 [17,18]	JULIET [19,20]	TRANSCEND [21,22,23]
**CRS (gr. ≥ 3)**	92% (11%)	58% (22%)	42% (2%)
**NT (gr. ≥ 3)**	67% (32%)	21% (12%)	30% (10%)
**Tocilizumab use**	43%	14%	19%
**Corticosteroid use**	27%	10%	21%

Axi-cel, axicabtagene ciloleucel; CRS, cytokine release syndrome; gr., grade; liso-cel, lisocabtagene maraleucel; NHL, non-Hodgkin lymphoma; NT, neurotoxicity; ref., reference; tisa-cel, tisagenlecleucel.

**Table 3:** Efficacy and toxicity data of selected ^§^ B-cell maturation antigen (BCMA)-targeted CAR-T-cell therapies in multiple myeloma (MM).

Trial Registry # [ref.]	N	ORR (CR)	Median PFS (*95% CI*)	CRS (gr. ≥ 3)	NT (gr. ≥ 3)
**NCT02546167** [38]	25	48% (8%)	NR	88% (32%)	32% (12%)
**NCT02215967** [39,40]	26	58% (8%)	NR	69% (23%)	4% (4%)
**NCT02658929** [41]	33	85% (45%)	11.8 mo (*6.2–17.8 mo*)	76% (6%)	42% (3%)
**NCT03090659** [42]	17	88% (82%)	12.2 mo (*NR*)	100% (41%)	0% (0%)
**NCT03090659** [43]	57	88% (74%)	15.0 mo (*11.0 mo-n.e.*)	89% (7%)	2% (0%)
**ChiCTR-17011272** [44]	21	95% (57%)	8.0 mo ^¶^ (*NR*)	91% (5%)	10% (NR)

BCMA, B-cell maturation antigen; CI, confidence interval; CR, complete response; CRS, cytokine release syndrome; gr., grade; MM, multiple myeloma; mo, months; N, number; n.e.: not estimable; NR, not reported; NT, neurotoxicity; ORR, objective response rate; PFS, progression-free survival; ref., reference. Trial registry #, study registration number on ClinicalTrials.gov (NCT#) or in the Chinese Clinical Trial Registry (ChiCTR-#); ^§^, only clinical studies published as full article in Web of Science/PubMed were selected (date of last search: 01 Jan 2020). ^¶^, PFS reported for patients with very good partial response or better.
